# Complete genome sequence of multidrug-resistant *Enterobacter roggenkampii* 0-E

**DOI:** 10.1128/mra.01149-23

**Published:** 2024-02-01

**Authors:** Jeff Gauthier, Irena Kukavica-Ibrulj, Laure Cockenpot, Vani Mohit, Jimmy Bernier, Chantal Brisson, Roger C. Levesque

**Affiliations:** 1 Institut de biologie intégrative et des systèmes (IBIS), Faculté de médecine, Université Laval, Québec, Canada; 2 Direction Générale de la coordination scientifique et du Centre d’expertise en analyse environnementale du Québec (DGCSCEAEQ); Ministère de l'Environnement, de la Lutte contre les changements climatiques, de la Faune et des Parcs (MELCCFP), Québec, Canada; Rochester Institute of Technology, USA

**Keywords:** antimicrobial resistance, enterobacteriaceae, carbapenems

## Abstract

Here, we present the complete 4.77 Mb genome of *Enterobacter roggenkampii* 0-E assembled with Oxford Nanopore long reads. This genome harbors 19 antimicrobial resistance genes, including *ramA* and *marA* decreasing permeability to carbapenems. This genome adds novel knowledge on emerging multidrug resistance in the *Enterobacter cloacae* species complex.

## ANNOUNCEMENT

*Enterobacter* spp. (1) cause a variety of foodborne infections in humans and livestock (2). In particular, resistance to carbapenems, a critical class of broad-spectrum antibiotics (3), appears widespread among *Enterobacter cloacae* complex strains (4). Furthermore, a multidrug-resistant *Enterobacter roggenkampii* isolated from Japan (OIPH-N260), also a member of the *E. cloacae* complex (5), possesses carbapenemase genes *bla_IMP-1_* and *bla_GES-5_* encoded on one IncP6 plasmid, thus placing *E. roggenkampii* as an emerging threat potentially capable of transferring carbapenem resistance to other pathogens (6). Here, we present the complete genome sequence and AMR profile of *E. roggenkampii* isolate 0-E.

*E. roggenkampii* 0-E was obtained from the Centre d’expertise en analyse environnementale du Québec and was grown at 37°C for 24 h on Brain Infusion Agar (BD Difco). One pure colony was resuspended in 2 mL Brain Infusion Broth (BD Difco) and grown at 37°C until it reached an OD_600_ of ~1.0. Cells from 1 mL culture were pelleted (14,000 × *g*, 5 min, 4°C), and genomic DNA was extracted using the DNeasy Blood and Tissue kit (QIAGEN) using the manufacturer’s recommended protocol for gram-negative bacteria.

Up to 1 µg of DNA was prepared with Oxford Nanopore’s Ligation Sequencing Kit (SQK-LSK109) and then sequenced on a GridION apparatus equipped with an R9.4.1 flow cell (FLO-MIN106). Bases were called *in situ* with MinKNOW 22.04 (REF), the GridION’s proprietary firmware. Prior to assembly, Nanopore reads were revised using the “correct” module from Canu v2.1 (7), leading to 32,862 consensus-corrected reads with an N50 of 13,7 kb. Then, assembly was performed with Unicycler v.0.5.0 (8). Medaka v1.4.3 (https://github.com/nanoporetech/medaka) was then used to polish the assembly. All software were run with default parameters unless specified.

The resulting assembly has 5.0 Mb length with a GC content of 55.5% and 26× mean coverage. It includes one chromosome (4,772,997 bp) and four plasmids of size 157,085, 62,568, 5,743, and 5,103 bp, respectively (Table 1). Annotation with PGAP version 20220210 (9) revealed 4,852 genes, of which 4,509 are protein coding.

**TABLE 1:** Main genome characteristics of *Enterobacter roggenkampii* 0-E^*a*^

Replicon	Size (bp)	CDS	tRNAs	rRNAs	AMR genes (abbreviations)
Chromosome	4,772,997	4,546	86	8	*MIR-10, FosA2, acrA, ramA, msbA, KpnE, KpnF, oqxA, adeF, marA, H-NS, baeR, emrR, emrB, rsmA, CRP, EF-Tu mutant, PBP3*
pER0-E1	157,085	168	–	–	no AMR gene detected
pER0-E2	62,568	66	1	–	*mcr-10.1*
pER0-E3	5,743	6	–	–	no AMR gene detected
pER0-E4	5,103	5	–	-	no AMR gene detected

^*a*^ CDS, coding DNA sequence; tRNAs, transfer RNA genes; rRNAs, ribosomal RNA genes; AMR, antimicrobial resistance. Gene annotations were produced with prokka v1.14.6 except for AMR genes which were annotated with RGI 6.0.1 web portal using the CARD v3.2.6 database.

Taxonomy was verified with GTDB-Tk v2.1.0 (10), which classifies prokaryotic genomes with both average nucleotide identity (ANI) and phylogenomic placement on a class-level tree. This genome was classified as *Enterobacter roggenkampii*, given 98.9% ANI to the closest reference (NCBI accession: GCF_001729805.1), which is also its nearest tree neighbor.

AMR gene annotation was done with Resistance Gene Identifier v5.2.0 (11) with CARD v3.0.6 as database (12) and revealed 18 putative chromosomal AMR genes and 1 on plasmid pER0-E2 (Table 1). From these chromosomal genes, two efflux genes (*ramA* and *marA*) are known to cause decreased permeability to carbapenems (13, 14). Their risk of horizontal spread, however, is unknown and requires further investigation of their genomic context. However, unlike *E. roggenkampii* OIPH-N260, no *sensu stricto* carbapenemase genes were detected.

## Data Availability

Raw reads and complete assemblies are available on the NCBI’s Genome and Sequence Read Archive (SRA) databases under the following BioProject number: PRJNA945021. The genome sequence is available under accession GCA_029536245.1; raw reads are available at SRR26902467.
